# Semiprone position is superior to supine position for paediatric endotracheal intubation during massive regurgitation, a randomized crossover simulation trial

**DOI:** 10.1186/s12871-018-0474-z

**Published:** 2018-01-18

**Authors:** Espen Fevang, Karin Haaland, Jo Røislien, Conrad Arnfinn Bjørshol

**Affiliations:** 1 Department of Research and Development, Norwegian Air Ambulance Foundation, Drøbak, Norway; 2 Department of Anesthesiology and Intensive Care, Stavanger University Hospital, Stavanger, Norway; 3 Department of Health Studies, University of Stavanger, Stavanger, Norway; 4 Department of Clinical Medicine, University of Bergen, Bergen, Norway

**Keywords:** Airway management, Intubation, Intratracheal, Emergency medical services

## Abstract

**Background:**

Endotracheal intubation of patients with massive regurgitation represents a challenge in emergency airway management. Gastric contents tend to block suction catheters, and few treatment alternatives exist. Based on a technique that was successfully applied in our district, we wanted to examine if endotracheal intubation would be easier and quicker to perform when the patient is turned over to a semiprone position, as compared to the supine position.

**Methods:**

In a randomized crossover simulation trial, a child manikin with on-going regurgitation was intubated both in the supine and semiprone positions. Endpoints were experienced difficulty with the procedure and time to intubation, as well as visually confirmed intubation and first-pass success rate.

**Results:**

Intubation in the semiprone position was significantly easier and faster compared to the supine position; the median experienced difficulty on a visual analogue scale was 27 and 65, respectively (*p* = 0.004), and the median time to intubation was 26 and 45 s, respectively (*p* = 0.001). There were no significant differences in frequency of visually confirmed intubation (16 and 18, *p* = 0.490) of first-pass success rate (17 and 18, *p* = 1.000).

**Conclusion:**

In this experiment, endotracheal intubation during massive regurgitation with the patient in the semiprone position was significantly easier and quicker to perform than in the supine position. Endotracheal intubation in the semiprone position can provide a quick rescue method in situations where airway management is hindered by massive regurgitation, and it represents a possible supplement to current airway management training.

## Background

Endotracheal intubation (ETI) is a procedure that is frequently performed both inside and outside hospitals [1]. When performed in an emergency setting outside the operating room, ETI differs from elective situations in several ways, with a relatively high complication rate [2]. One of the most frequent causes of difficulties concerning ETI in the pre-hospital setting is the presence of blood, vomit, debris and secretions blocking the view and disturbing laryngoscopy [3]; these factors affected 49% of all attempted pre-hospital ETIs by experienced trauma anaesthetists in one article [4]. Videolaryngoscopic devices have shown promising results when performing ETI in patients with a difficult airway, but, like conventional laryngoscopes, these devices are dependent of an unobscured view to work properly [5–7]. Portable suction units are often available for emergency ETI, but they are not always readily accessible on-scene and are sometimes insufficient, especially in the presence of gastric contents that have a tendency to block the suction catheters [8]. Methods and equipment that can be used during massive regurgitation have been described [9–11], but despite a relatively frequent occurrence, few publications exist.

An alternative technique was successfully improvised after an accident in our district. Here, two children aged three and five had confirmed asystole and fixed dilated pupils after a water submersion time of approximately 11 and 13 min, respectively. All ventilation and intubation attempts in the supine position were impossible because of massive regurgitation with a mixture of gastric contents, seawater and pulmonary fluids, and the available manual suction unit was instantly blocked by food solids. Drainage in the recovery position with subsequent return to the supine position was attempted, but the regurgitation was so severe that the oral orifices were refilled immediately before any airway management could be attempted. By keeping the patients in the overturned position, gastric and pulmonary contents drained constantly, without blocking the laryngeal view. Identification of the laryngeal opening was facilitated by bursts of pulmonary fluid during chest compressions, and visually confirmed intubation of both patients was uncomplicated in this position. Both children underwent ETI in less than two minutes after water extrication, and there were no interruptions in chest compressions during the procedure. Return of spontaneous circulation occurred in both patients within 20 min after cardiopulmonary resuscitation (CPR) started, and both patients were discharged from the hospital with no known sequelae.

Although performing ETI in positions that allow for drainage seems quite intuitive, the available literature on this is scarce. Some articles address intubation in various lateral positions [12–14], but this differs from the method used here, which was closer to a prone position, where both anatomy and drainage are affected in a different way. However, one article had very promising results after routine intubation in the prone position [15], and ETI in the lateral recovery position has been recommended for post-tonsillectomy haemorrhage in textbooks [16].

The aim of this study was to examine whether turning the patients over to the semiprone position during ETI is superior to a regular ETI in the supine position, as a rescue method when massive regurgitation occurs. Primary endpoints were experienced difficulty in performing ETI in each of the two positions, as measured on a visual analogue scale (VAS) [17], and time to successful ETI, measured in seconds. Secondary endpoints were frequency of self-reported visually confirmed intubation and ETI-success on the first attempt.

## Methods

### Study design

This simulation study was conducted as a crossover trial, with participants randomly assigned into two groups. Eighteen anaesthesiologists with different levels of experience performed ETI twice: once in the supine position and once in the semiprone position.

**Fig. 1:**
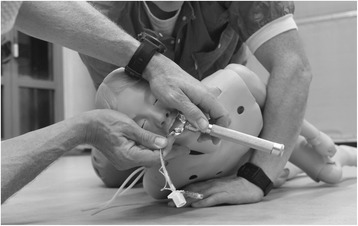
Laryngoscopy in the semiprone position

**Fig. 2:**
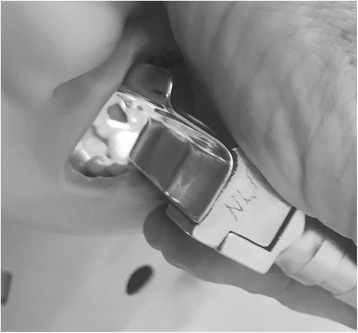
Laryngeal view during laryngoscopy in the semiprone position. The position allows for constant drainage from the oral orifice throughout the procedure

In a strict *lateral position*, the body is turned approximately 90 degrees away from the supine position, and gravitational forces on the anatomic structures result in a lateral shift of the tongue, uvula and surrounding tissues, disturbing what is considered to be a normal view when compared to the supine position [13]. Our alternative, a *semiprone position*, involves turning the patient further towards the prone position, to above approximately 125 degrees (Fig. 1). By turning the patient this far, the largest anatomical structures in the mouth start to fall directly away from the laryngeal opening, with a possible normalisation of the view, allowing for laryngoscopy and ETI with simultaneous drainage from the oral orifice (Fig. 2).

To minimize learning bias, half of the participants began with ETI in the supine position followed by ETI in the semiprone position; the other half began and ended with the converse positions. Participants were divided randomly into the two groups using randomizer.org [18]. All participants were given a demonstration of the procedure before performing ETI in the semiprone position on an unsoiled Laerdal Airway Management Trainer (Laerdal Medical, Stavanger, Norway).

### Simulation setup

For the simulation experiment, we used a SimJunior® manikin (Laerdal Medical, Stavanger, Norway). All experiments started with the manikin placed on the floor in the supine position, as the method is meant to be used as a rapid rescue method that can be applied when regurgitation occurs. A laryngoscope with Macintosh blade number two and a cuffed tube size 4.0 (Rüshelit® Super safety clear, Teleflex Inc., Wayne, Pennsylvania, USA) was used for ETI. A manual suction unit identical to the one used in our pre-hospital service (RES-Q-VAC®, RMS medical products, Chester, New York, USA) with an 18 CH/53 cm suction catheter (Mediplast AB, Malmö, Sweden), operated by an assistant, was available during the entire experiment. As we wanted to investigate a real-life situation where the available equipment is limited, a standard and readily-available flexible, rather than a potentially more effective rigid suction unit was used in the simulation study.

To avoid drainage from the oropharynx into the manikin during the experiment, the oesophagus was clamped below the larynx without affecting the upper airway. Regurgitation was simulated by filling the oral orifice up to the frontal teeth with a porridge-like canned stew with a typical bit-size of 7 mm (X-tra Brun Lapskaus, Norway), heated to body temperature and diluted with half its volume of water. To simulate continuous inflow, a 14 G intravenous catheter (14G × 45 mm BD Venflon™ Pro Peripheral IV catheter, Franklin Lakes, New Jersey, USA) was placed in the oesophagus above the clamp and was connected to a closed pressure infuser containing a bag of 1000 NaCl 0.9% at 300 mmHg with a measured flow of 250 ml/min. Participants were instructed to manage the airways such as they would have done in a real-life situation with the tools available. When performing ETI in the supine position, the manikin could be turned over for drainage at the participant’s request, but the actual ETI had to be done with the manikin flat on its back. For the semiprone position, the manikin was consistently turned to the right during this experiment, as we found that this leads to more free movement with the left hand holding the laryngoscope (Fig. 1). Before starting, the participants kneeled on the floor above the manikin, placed in the supine position, with all necessary equipment within reach. An assistant was available at their left side, providing help with suctioning, positioning, and support of the manikin’s head, at the participant’s request.

### Measurements

The primary endpoints were the experienced difficulty of the intubation, assessed using a VAS-scale from 0 mm (extremely easy) to 100 mm (extremely difficult), and the time to a confirmed successful intubation in seconds. At the beginning of the experiment, a timer was started, and the pressured NaCl was opened fully. The definition of a successful intubation was a correctly placed tube in the manikin’s trachea, controlled with bag ventilations and visual conformation by the observer after the attempt was discontinued. The time was recorded when the tube was considered by the participant to be correctly placed. In the case of an incorrectly placed tube on examination, the total time after subsequent attempts were noted. The VAS scores for experienced difficulty was noted by the participants themselves immediately after ETI in both positions had been completed. The frequency of self-reported visually confirmed intubation, and the number of intubation attempts, were noted by the observer immediately after the experiment. Level of experience amongst the participants were categorized into the following four categories based on the self-reported estimated number of performed intubations: < 50, 50–200, 200–2500 and > 2500.

### Power calculation

To the best of our knowledge, the minimal clinically important time difference between emergency airway management methods is yet to be addressed in the literature. In cardiac arrest patients, however, there is consensus that all hands-off time (time without chest compressions) must be minimized, and existing guidelines state five seconds as the maximum hands-off-time [19–22]. As patients in cardiac arrest are likely to be amongst those with massive regurgitation during airway management, we used this number in our study size calculation [23]. Performing a pilot study on time-to-intubation in five persons, we found a standard deviation (SD) in difference in intubation time of 6.2 s. Using this SD, we estimated that 12 participants were necessary to achieve 80% power and a *p*-value of 5% for detecting a difference of 5 s for ETI in the two positions. To allow for possible failed intubations and other unforeseen adverse events, we added another six participants (50%), resulting in a total number of 18 participants.

### Statistical analysis

Continuous data were summarized using the median (range) and compared using related-samples Wilcoxon signed rank tests, because data were not normally distributed. Binary data were summarized as frequencies and compared with Fischer’s exact tests. A *p*-value of < 0.05 was considered statistically significant. Statistical analysis was performed using IBM SPSS Statistics 23.0 (IBM Corp. Released 2015. IBM SPSS Statistics for Windows, Armonk, NY: IBM Corp.).

## Results

All participants managed successful ETI in both positions. One participant needed two attempts in the supine position. Two participants did not achieve visual confirmation of the intubation in the supine position; all had visual confirmation in the semiprone position. Of the 18 participants, one had performed a total of < 50 intubations, three had 50–200 intubations, seven had 200–2500 intubations, and seven had > 2500 intubations. The actual insertion of the tube in the semiprone position was considered to be a potential problem during the simulation, but this turned out to be uncomplicated. The participants were told to do what they felt was the most intuitive, and no participants hesitated at this point. The assistant supported the head during the intubation in most cases, while for some the person performing the intubation supported the head with the inside of the hand, while digits one and two were used to insert the tube (Fig. 1).

**Fig. 3:**
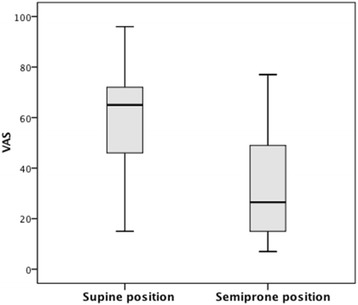
Experienced difficulty of intubation with the manikin in the two different positions, measured on a Visual Analogue Scale (VAS)

**Fig. 4:**
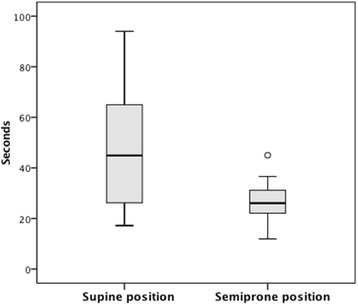
Time to intubation (seconds)

VAS for experienced difficulty in ETI in the two positions are summarized in Fig. 3. In the supine position, ETI was significantly more difficult than in the semiprone position, with a median (range) VAS of 65 (15–96) versus 27 (7–77), respectively (*p* = 0.004). Time to intubation for the two positions are summarized in Fig. 4. Time to intubation was significantly longer in the supine position, with a median (range) of 45 (17–94) seconds versus 26 (12–45) seconds, respectively (*p* = 0.001).

There were no statistically significant differences for the secondary endpoints. There were 16 (89%) and 18 (100%) visually confirmed intubations for supine and semiprone positions (*p* = 0.490), respectively, and 17 (94%) and 18 (100%) successes in the first attempt for supine and semiprone positions, respectively (*p* = 1.000).

## Discussion

In our experiment, ETI in the semiprone position was superior to ETI in the supine position for both primary endpoints. A correctly positioned cuffed endotracheal tube remains the gold standard for definitive airway management [24–26], and we find the results from our simulation study promising. Few publications have examined solutions to situations where regurgitation complicates airway management, and we believe our proposed simple technique may represent an important supplement to airway management training. After having the method demonstrated to them just once, the participants in our study were able to handle a highly challenging airway management situation, where all participants achieved a visually confirmed intubation in the semiprone position within a short period of time. Although the time difference between the two positions of 19 s may not seem crucial in a controlled in-hospital setting, in the case of an emergency situation with an uncontrolled airway, all extra time spent can be of great importance, and the result is well above the suggested clinically important time difference of five seconds [19].

Given the simplicity of the intervention, we hypothesized that the method might be relevant for all personnel performing intubations at a regular basis. We therefore included personnel with different levels of experience in the experiment. Although the clinical experience of the participants was heterogeneous, the results were surprisingly consistent and homogeneous. Only two participants found the semiprone position more difficult; both participants were highly experienced, with > 2500 performed intubations. Among the less experienced personnel, all found ETI in the semiprone position easier during simulated regurgitation. We believe that this might imply that the technique can be relevant also for personnel with less extensive airway management experience.

Some patients can be expected to be in cardiac arrest during an actual situation, and the quality of CPR may be affected by turning the patient away from the supine position. Effective chest compressions in the lateral position have been described in the literature, but evidence is limited [27]. In the incident described in the introduction, chest compressions by a well-trained health professional with a depth of the recommended approximate five centimetres could be continued in the semiprone position by placing one hand on the chest and the other on the child’s back; this would probably not have been easily achieved in an adult patient [27–29]. However, our main aim was to examine the position’s effect on the possibility of performing ETI, and the experiment was conducted without chest compressions. We do not know how positioning affects airway management during continuous chest compressions, but interruptions in CPR during all invasive airway management are well documented [30, 31]. The need for interruptions during ETI in the semiprone position does not necessarily differ widely from other alternatives, and the shortened time spent on the procedure may be of possible benefit.

During turning of unconscious trauma patients, neck injuries are of concern, and the presence of a neck collar or suspected neck injury might interfere with intubation and the overall situation. Manual stabilization during a log roll and ETI in the semiprone position is not impossible, but it is difficult, and it calls for more trained personnel; a scarce resource in the pre-hospital setting. In a real-life setting with both a suspected neck injury and a flooded airway, the clinician will have to make a decision based on a critical evaluation of which risk outweighs the other.

Our result suggests that turning a paediatric patient over to the semiprone position can represent an immediate rescue method in situations where ETI is complicated by vomit, blood or other foreign matter blocking the laryngeal view, and we believe intubation in the semiprone position should be considered before conducting a tracheostomy. Being a simulation study, the results should be interpreted with caution. However, the method has been used in real-life situations where normal attempts for intubation failed, with immediate success. In the case of sudden regurgitation, the method can be performed immediately, without any need for equipment or extra preparation, and the position gives some protection from aspiration until the airway is secured [32]. In cases where ETI in the semiprone position fails, return to the supine position can be performed within seconds. The cost of training is minimal, and we believe that all personnel likely to encounter difficulties with airway management resulting from foreign matter may benefit from practicing ETI in the semiprone position on a manikin.

### Limitations

This is a simulation study and thus holds several limitations. Although the experiment attempted to re-create a real clinical setting in which the technique was used, this can never be completely accomplished, and both the surroundings and the use of a manikin with no dynamic components may have affected outcome. The experiment could not be performed with any blinding, so there is risk of bias as the participants may have had a personal expectation of intubation in the semiprone position to be superior after the initial demonstration. Although the simulation was experienced as difficult, it was performed in a favourable setting, with necessary equipment available, good lighting conditions, regular room temperature and enough space to allow for necessary movement. All participants perform ETI on a regular basis, and they are likely to be more comfortable with the procedure than personnel that only rarely do it. Further, we did not ask for any additional treatment, such as mask-bag ventilations, CPR or intravenous cannulation. Addition of such real-life considerations could change the degree of difficulty and time to successful ETI.

Both the described incident and the simulation were performed on paediatric patients and manikins with body weights below 20 kg. Small patients are normally easily turned over and a transition of our findings from paediatric to adult patients is not necessarily valid. However, in real-life situations, the main author has experienced success with ETI in the semiprone position on regurgitating adult patients with body weights above 100 kg.

## Conclusions

In this simulated situation with massive regurgitation in a paediatric manikin, ETI in the semiprone position was significantly faster and easier to perform when compared to the supine position. There were no significant differences in the rate of successful ETI or number of intubation attempts between the two positions.

## Abbreviations

CPR: Cardiopulmonary resuscitation
ETI: Endotracheal intubation
SD: Standard deviation
VAS: Visual analogue scale
